# Pityriasis Rubra Pilaris Following COVID-19 Infection: A Case of Successful Treatment With Ixekizumab

**DOI:** 10.7759/cureus.78668

**Published:** 2025-02-07

**Authors:** Madelyn N Ross, William T Snider, Ian Depew, Shane Cook

**Affiliations:** 1 Department of Dermatology, Marshall University Joan C. Edwards School of Medicine, Huntington, USA

**Keywords:** covid-19, il-17 inhibitor, ixekizumab, pityriasis rubra pilaris, psoriasiform dermatoses

## Abstract

Pityriasis rubra pilaris (PRP) is a rare dermatologic disorder marked by erythema, scaling, pruritus, pain, and follicular hyperkeratosis. It usually appears as red, scaly patches with areas of unaffected skin and is commonly associated with orange-colored palms and soles. While the exact etiology is unknown, emerging evidence points to several contributing factors. PRP can vary in severity, ranging from localized lesions to extensive erythroderma involving nearly the entire body surface. This report details the case of a 74-year-old Caucasian male who presented with a four-month history of an erythematous, pruritic rash affecting the face, trunk, and extremities. The rash developed following a COVID-19 infection, complicating the diagnostic process. A skin biopsy confirmed psoriasiform dermatitis consistent with PRP. This case focuses on the diagnostic challenges of PRP, particularly following a COVID-19 infection, and explores the potential role of IL-17 inhibitors in its treatment.

## Introduction

Pityriasis rubra pilaris (PRP) is a rare dermatologic disorder characterized by erythema, scaling, and follicular hyperkeratosis, with an estimated incidence of approximately 1 in 5,000 dermatologic patients [1]. The condition is classified into six subtypes, which vary based on the age of onset and clinical presentation. Type I, the most common subtype, typically manifests with erythematous papules that merge into plaques, leaving areas of unaffected skin [2]. Histologically, it is characterized by a distinctive checkerboard pattern of hyperkeratosis [2]. While the exact etiology remains unclear, PRP has been linked to genetic, immune, and environmental factors, and recent research suggests a role for immune dysregulation in its pathogenesis [2]. In addition, emerging evidence indicates that COVID-19 infection and vaccination may serve as potential triggers for the onset of PRP, highlighting the need for clinicians to consider these factors when diagnosing the condition [1].

Patients with PRP can present with symptoms ranging from mild forms affecting only the extensor surfaces to severe erythroderma covering 90% to 100% of the body, accompanied by intense itching, skin tightness, and pain [3]. Diagnosing PRP can be challenging due to its overlap with other dermatologic conditions, requiring histopathological confirmation. While some cases resolve on their own, chronic symptomatic cases need ongoing treatment, and biologics targeting the IL-17/IL-23 pathway are emerging as a promising option [3]. We present a case of PRP that initially began treatment with topical tacrolimus and clobetasol. After an inadequate response, therapy was escalated to acitretin. Following the failure of these treatments, the patient transitioned to ixekizumab injections, which have resulted in mild improvement. This case highlights the complexities involved in managing PRP and emphasizes a successful case of PRP treated with ixekizumab and topical clobetasol.

## Case presentation

**Figure 1:**
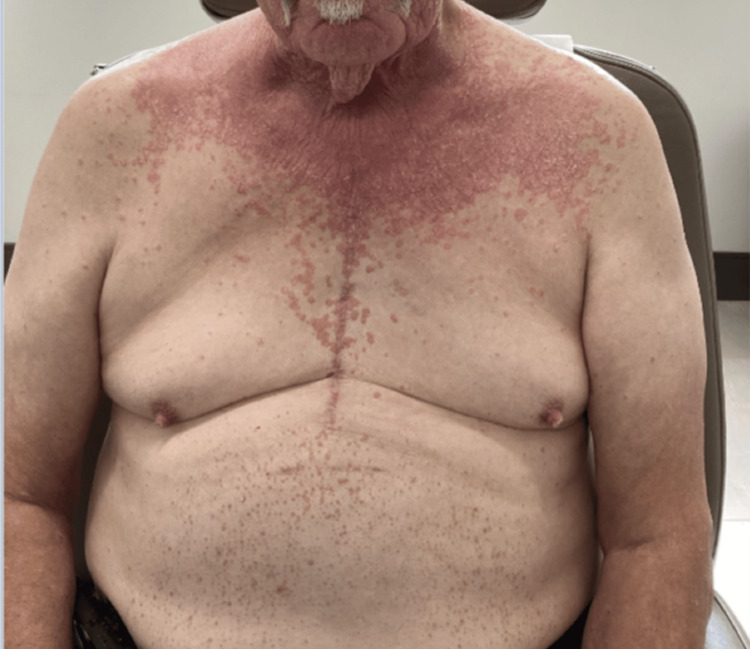
Erythematous papules coalescing into plaques on the anterior chest

**Figure 2:**
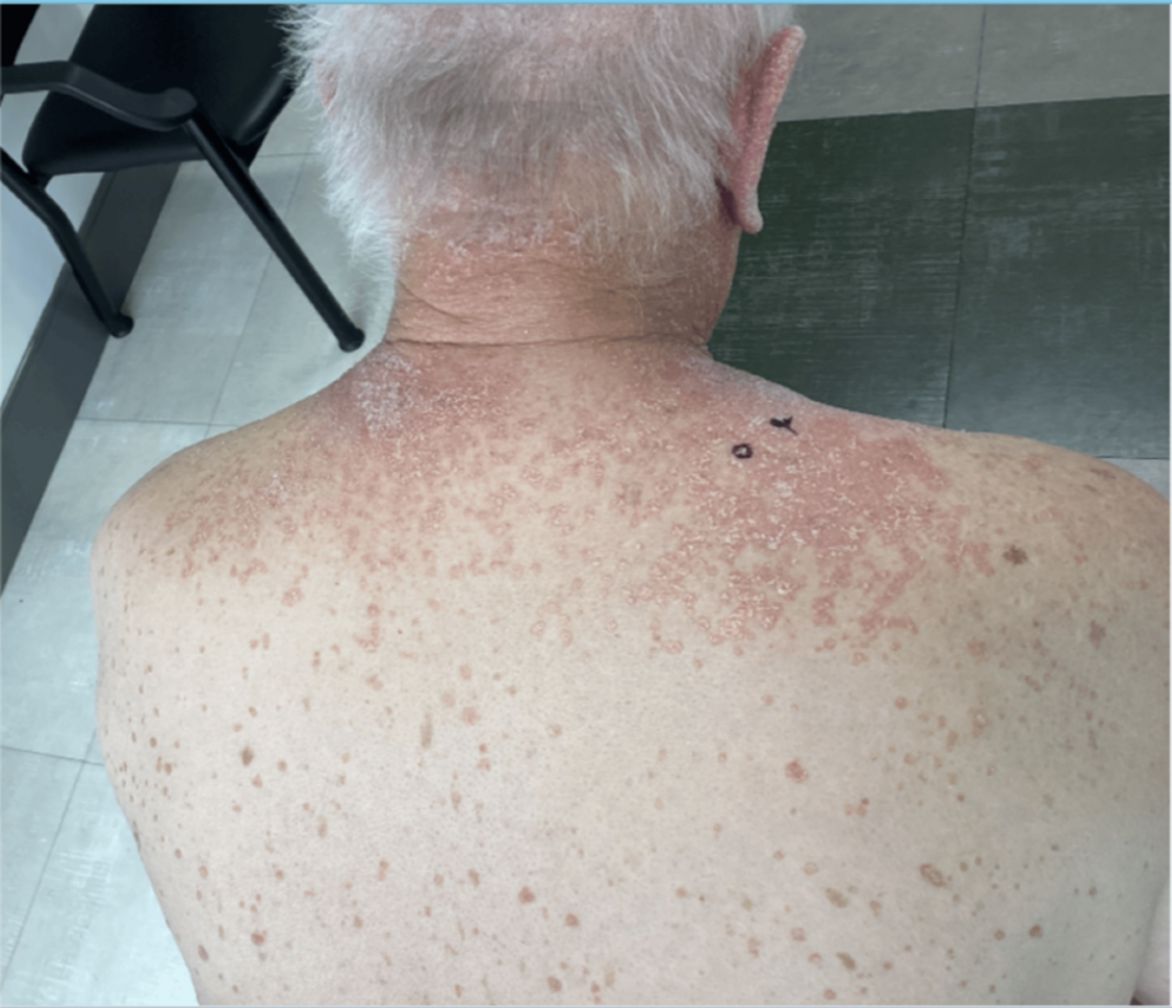
Erythematous papules and plaques on the upper back and neck

**Figure 3:**
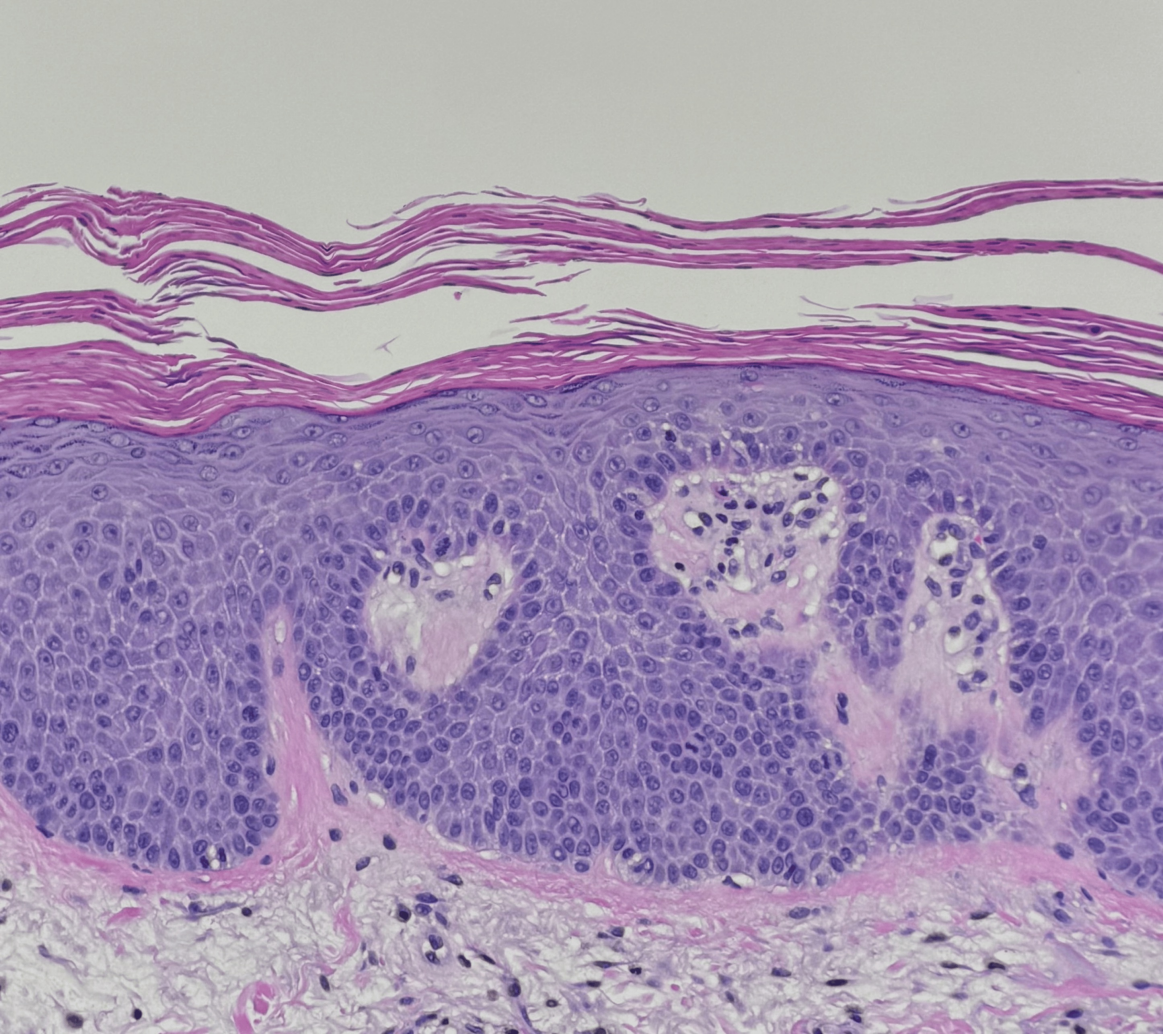
Histopathological findings showing follicular plugging and alternating parakeratosis, hallmarks of PRP PRP: Pityriasis rubra pilaris

A 74-year-old white male initially presented with a four-month history of a red, itchy rash involving the face, arms, chest, and back (Figures 1-2). The rash developed following a COVID-19 infection. Exam findings revealed psoriasiform papules coalescing into plaques on the upper back, chest, and face, along with salmon-colored plaques on the palms. Differential diagnoses at the time included PRP, psoriasis, cutaneous T-cell lymphoma, and systemic lupus erythematosus. A skin biopsy of the upper back showed psoriasiform epidermal hyperplasia with focal spongiosis, hyperparakeratosis with alternating orthokeratosis, and plugged follicles with shoulder parakeratosis (Figure 3). The physical exam and histopathology findings were consistent with PRP.

Initial treatment included topical tacrolimus for the face and clobetasol for the trunk and hands. After failing this initial treatment, the patient was started on acitretin 25 mg. Four weeks after starting acitretin, the patient reported worsening of the rash. Examination revealed increased body surface area involvement, including the face, trunk, and arms, along with persistent, orange-colored palms. Acitretin was discontinued, and the patient was transitioned to ixekizumab 80 mg every two weeks. At the four-week follow-up, there was a slight improvement in the coloration and scaling.

## Discussion

Beyond the challenge of diagnosing PRP, treating it is even more difficult. PRP is often resistant to treatment, with no current US Food and Drug Administration-approved therapies available. This is partially due to the fact that the underlying mechanism of PRP has not yet been fully established. It is thought to result from genetic factors, disruptions in vitamin A metabolism, altered immune response to superantigens, infections, and other triggers, including drug exposure, malignancies, and autoimmune diseases [4]. Recent studies suggest that dysregulation of the interleukin-23/T-helper-17 cell axis plays a key role in PRP pathogenesis [2].

Recent research indicates a potential connection between PRP and COVID-19 infections, with some patients developing PRP symptoms following a viral infection, which adds complexity to understanding its underlying mechanisms. In a scoping review by Zhou et al. (2024), which analyzed 221 studies, the authors identified common triggers for PRP-like eruptions or PRP diagnosis. The most frequently reported factors were COVID-19 vaccination (12 cases), malignancy leading to paraneoplastic PRP (10 cases), and COVID-19 infection (5 cases) [1]. Although the exact pathophysiology of PRP remains unclear, the review underscores the importance of considering COVID-19 infection and vaccination as potential triggers when diagnosing PRP.

Treatment options for PRP vary widely, ranging from initial topical treatments to systemic oral therapies. Topical treatments for PRP include corticosteroids, emollients, keratolytics, and topical tretinoin, which are used for localized cases or in combination with systemic therapies for more severe forms [4]. Systemic treatments include retinoids, methotrexate, or phototherapy; biological agents targeting the IL-17/IL-23 pathways have shown promise for refractory cases [4]. New therapies, such as IL-17 inhibitors (Secukinumab, ixekizumab) and IL-23 inhibitors (Risankizumab, guselkumab), are emerging as potential treatments for PRP [4].

Abduljawad et al. (2023) conducted a systematic review of 19 articles involving 77 cases, evaluating the effectiveness of IL-17 inhibitors (Secukinumab and ixekizumab) in treating PRP [5]. The findings showed that both biologics were highly effective, with secukinumab achieving nearly 90% complete improvement, particularly in types 1, 3, and 4 PRP [5]. Ixekizumab also demonstrated significant results, though with a lower response in patients with unknown PRP types [5].

IL-17 inhibitors, particularly secukinumab, are well-recognized as effective treatments for PRP. While secukinumab has seen widespread use for this condition, ixekizumab, another IL-17 inhibitor, has been less commonly utilized. In this case, due to the patient being a VA patient and secukinumab not being available on the formulary, ixekizumab was chosen for treatment. This highlights the broader potential of IL-17 inhibitors, suggesting that both secukinumab and ixekizumab, as part of the same class, are likely effective options for managing PRP, offering flexibility in treatment strategies. This case demonstrates the success of biologics, specifically IL-17 inhibitors, and their promising role in treating PRP. While not yet FDA-approved, biologics are emerging in the literature as a potential treatment for PRP.

## Conclusions

This case emphasizes the challenges of diagnosing and managing PRP, a rare and complex dermatologic condition. This patient’s disease progression despite first-line treatments illustrates the variability in treatment responses and emphasizes the necessity of a personalized, case-by-case management approach. The patient had a recent COVID-19 infection prior to presenting with the rash, suggesting a potential link between the virus and the development of PRP. Emerging biologics, such as IL-17 inhibitors including ixekizumab, present a promising treatment option. Early recognition and diagnosis are essential for delivering appropriate therapy and improving patient outcomes. Ultimately, this case highlights the importance of maintaining awareness and adaptability when managing chronic, severe dermatologic conditions such as PRP.
